# Gender Equality, Drinking Cultures and Second-Hand Harms from Alcohol in the 50 US States

**DOI:** 10.3390/ijerph16234619

**Published:** 2019-11-21

**Authors:** Katherine J. Karriker-Jaffe, Christina C. Tam, Won Kim Cook, Thomas K. Greenfield, Sarah C.M. Roberts

**Affiliations:** 1 Alcohol Research Group, Public Health Institute, Emeryville, CA 94608, USA; ctam@arg.org (C.C.T.); wcook@arg.org (W.K.C.); tgreenfield@arg.org (T.K.G.); 2 Advancing New Standards in Reproductive Health (ANSIRH), Department of Obstetrics, Gynecology & Reproductive Sciences, University of California, San Francisco, Oakland, CA 94612, USA; sarah.roberts@ucsf.edu

**Keywords:** alcohol’s harms to others, gender equality, drinking cultures

## Abstract

Background: Gender inequality and cultures of binge drinking may increase the risk of second-hand harms from alcohol. Methods: Using the 2014–2015 National Alcohol Survey and 2015 National Alcohol’s Harm to Others Survey (N = 7792), we examine associations of state-level gender equality measures (contraceptive access, abortion rights, women’s economic equality) and binge drinking cultures (rates of men’s and women’s binge drinking) with individual-level indicators of second-hand harms by drinking strangers and partners/spouses. Results: In main effects models, only male binge drinking was associated with greater odds of harms from drinking strangers. There were significant interactions of gender equality with male binge drinking: High male binge drinking rates were more strongly associated with stranger-perpetrated harms in states low on contraceptive access or abortion rights compared to states high on these measures. Conversely, male binge drinking was more strongly associated with spouse/partner-perpetrated second-hand harms in states with more economic equality, compared to states lower on this measure. Conclusions: Detrimental effects of high male binge drinking rates may be modified by gender equality. Targeted interventions may reduce alcohol-related harms experienced by women in states with high rates of male binge drinking. Restrictions in access to contraception and abortion may exacerbate harms due to men’s drinking.

## 1. Introduction

Gender equality refers to the parity of women and men (an absence of inequity or bias) on key economic, social and political indicators [1]. Gender inequality is rooted in the power structures in a society that allocates resources and opportunities differentially to men [2]. Globally and in the United States (US), gender (in)equality has been linked to many health outcomes, particularly for women [3,4] and children [5,6], but also for men [4,7]. For example, in the US, greater state-level gender equality is associated with better mental health for women [3], reduced teen pregnancy [6], better birth outcomes [6], reduced infant mortality [5] and lower mortality rates for both men [4,7] and women [4]. Gender equality encompasses dimensions of economic participation and opportunity (such as the proportion of women engaging in paid labor), educational attainment (such as the proportion of women with a university education), political participation and empowerment (such as the proportion of women who voted in a recent election or the proportion of women in state-wide elected office), and reproductive rights (such as access to abortion services) [8,9,10,11,12], and each of these dimensions may contribute to better health.

Greater gender equality may be associated with increases in some health risk behaviors among women [2]. This could be due to a combination of changes in social norms, industry tactics to increase market share [13] and a variety of other factors such as women’s disposable income and leisure time [14]. There is some concern that greater gender equality may be associated with increased alcohol consumption among women [15,16]. In the US, data do not appear to support concerns about greater gender equality leading to increased drinking by women, however. One US study found women’s drinking was either negatively or not significantly associated with state-level gender equality measures including reproductive rights, women’s political participation and gender equality in socioeconomic status (SES) [12]. In fact, findings suggested that increased gender equality was associated with lower levels of risky drinking by both women and men [12]. The current study examines associations between gender equality and second-hand harms from alcohol, including an investigation of the moderating effects of men’s and women’s drinking cultures.

Second-hand harms due to alcohol—which also are called alcohol’s harm to others, externalities and collateral damage from drinking [17]—range in severity from complaints, such as being kept awake at night by drunken noise in the streets, or being harassed or bothered by someone who had been drinking, to more severe concerns, such as being assaulted by someone who had been drinking or being in a drink-driving accident [18]. These second-hand effects of alcohol are prevalent. US general population survey data suggest almost 20% of adults report at least one harm caused by someone who had been drinking in the prior year [19], and lifetime estimates of these harms approach 60% [20]. Rates of harm are even higher in other countries such as Canada [17] and Australia [21], as well as in some low- and middle-income countries [22]. There also is notable geographic variability within societies in the prevalence of second-hand harms from alcohol [22], as well as in the social patterning of these harms [23]. For example, recent US data show that women report more harms from drinking spouses/partners compared to men [19,24,25], with an elevated risk of physical aggression from such perpetrators [25]. These second-hand effects of alcohol have serious consequences for mental health [19,26,27,28,29], particularly if the harms were caused by someone close to the victim, such as a drinking spouse or family member [19,27,28].

Many studies have examined the relationship between gender equality and violence against women (see, for example [30] and the review by Roberts [8]), but fewer have looked specifically at alcohol-related harms. A US study using neighborhood (small area) data found greater gender equality was associated with reduced rates of violence perpetrated by young men, with no increases in violence perpetrated by young women [31]. An international study found gender equality was associated with smaller differences between women’s and men’s drinking in public places, such as bars [32]. Those authors speculated that an increased presence of women in these public drinking venues could put them at increased risk of alcohol-related harm. They also posited, alternatively, that the increased presence of women also could change the culture of public drinking venues by making the places less masculine, thus perhaps reducing alcohol-associated harms, such as fights or other violent events that often occur in such locales. They actually found the opposite in a subsequent paper [33], with a decreased presence of women in bars in some countries appearing to be protective for men in terms of reducing fights. However, this study focused only on the proportion of men and women drinking in bars as the key predictor, and it included a range of countries that varied in terms of drinking culture (including rules and norms about the acceptability of drinking) and other factors, such as rates of violence, that could not be adequately controlled in analyses. Here, we focus on whether state-level indicators of gender equality in the US are associated with second-hand harms due to someone else’s drinking, as well as the ways in which drinking cultures may affect this association.

Significant differences across cultures in people’s attitudes, norms and behaviors related to drinking have been noted [34]. In addition to varying in gender equality, the 50 US states also vary in men’s and women’s drinking patterns [35,36], foretelling variability in drinking cultures by gender. In the US, each state has unique characteristics relevant to the determination of a drinking culture and to corresponding alcohol consumption patterns and alcohol-related problems. 

Factors that may underlie or influence drinking culture include a state’s demographic make-up in terms of age, gender, race/ethnicity groups, and socioeconomic status; a state’s mix of religions with differing perspectives on alcohol use; historical and cultural practices related to drinking (or not drinking); and state alcohol policies and regulatory regimes [35]. There are indications that state binge drinking rates are a reasonable proxy for drinking culture, and that these may influence drinking behaviors, as well as the second-hand effects of drinking. For example, a US study found the state binge drinking rate was an independent predictor of binge drinking among students who attended college in the same state [37]. To date, we are unaware of any published studies that have examined state binge drinking rates in relation to second-hand harms from drinking, although one study found proximity to colleges with high levels of binge drinking was related to greater reports of second-hand harms by community members living in nearby neighborhoods [38]. This suggests that risks for second-hand harms perpetrated by drinking strangers may be elevated in states with high rates of binge drinking. Second-hand harms caused by known drinkers also may be elevated in such states. This may be particularly true for female victims, as women are more likely than men to report being close to a male heavy drinker [39] (e.g., a spouse/partner) and women in many countries—including the US, Australia and New Zealand—are more likely than men to experience harm caused by a known drinker [23].

Based on the extant literature showing associations between gender equality, alcohol consumption and violence, we hypothesize that harms due to someone else’s drinking would be more common in states with lower gender equality (Hypothesis 1a) and in states with high rates of binge drinking (Hypothesis 1b), especially binge drinking by men. We also examined whether these contexts had synergistic effects, anticipating that the combination of low gender equality with higher rates of men’s binge drinking would be associated with particularly elevated levels of second-hand harms from alcohol. That is, we hypothesize that the expected relationship between a culture of binge drinking by men in the state and second-hand harms due to drinking would be exacerbated by gender inequality (Hypothesis 2). Given gender differences in the social patterning of second-hand alcohol harms, we also expected the interaction of gender inequality and drinking culture to emerge for alcohol-related harms reported by women rather than for harms reported by men (Hypothesis 3). For men, we instead anticipated that higher rates of binge drinking would be associated with more harms, regardless of the level of gender equality in the state. Because we were not sure how these associations might differ by perpetrator type, we examined an overall indicator of harms, and then used separate indicators for stranger- and spouse/partner-perpetrated harms.

## 2. Materials and Methods

### 2.1. Survey Datasets

We used pooled cross-sectional data from the 2014–2015 National Alcohol Survey (NAS) and 2015 National Alcohol’s Harm to Others Survey (NAHTOS). These surveys incorporated the same dual-frame landline and mobile telephone sampling design that also included Black/African American and Hispanic/Latino oversamples (hereafter Black and Hispanic, respectively). One eligible respondent from each household was randomly selected, and computer-assisted interviews were administered in either English or Spanish. The overall cooperation rates for the NAS and NAHTOS were 45.4% and 47.3%, respectively (averaged across landline and cell phone surveys). The Institutional Review Boards of the Public Health Institute in Oakland, CA, and of the fieldwork agency (ICF, Inc., Fairfax, VA, USA) approved all survey protocols. The human subjects’ protections for the 2015 US National Alcohol Survey were approved on 6 September 2013 (IRB#I13-019). The protocol was subsequently included in an overarching approval (with ongoing, yearly oversight) for the National Alcohol Survey Series (IRB#I16-028). The human subjects protections for the 2015 US National Alcohol’s Harm to Others Survey were approved on 18 December 2014 (IRB#I13-018). For more details on the NAS, see Karriker-Jaffe et al. [19], and for more information on the NAHTOS, see Kaplan et al. [40]. 

For both surveys, respondent addresses were geocoded and linked with the state-level predictors described below. Due to missing data on some state-level indicators, respondents living in Washington, DC (*n* = 49) were not included in the analyses.

The analytic sample (*N* = 7792) was restricted to respondents who had no missing data on key dependent variables, and who had location identifiers at the state level. In the weighted sample, 52.6% of respondents were female (*n* = 4636), the average age was 47.1 years old (SD = 17.8) and the majority was non-Hispanic White (66.3%), followed by Hispanics (14.1%), Blacks (11.9%) and respondents of other or missing race/ethnicity (7.7%).

### 2.2. Measures

#### 2.2.1. Outcome Variables

Respondents reported whether they experienced any of eight harms due to someone else’s drinking in the past year. Second-hand harms included: (a) having family problems or marriage difficulties; (b) being pushed, hit or assaulted; (c) having house, car or other property vandalized; (d) having financial trouble; (e) being in a traffic accident; (f) being harassed, bothered, called names or otherwise insulted; (g) feeling threatened or afraid; and (h) being physically harmed. These items have been used on prior US [19,20,41], Canadian [42] and multi-national surveys [43]. As previously detailed [19], each harm was attributed to specific perpetrators, including intimate partners (spouses as well as boyfriends/girlfriends) and/or strangers (someone not known by name). In the analytic sample, 19.2% had experienced one or more of these harms in the past year, with 4.0% reporting harm due to the drinking of intimate partners and 8.8% reporting second-hand harm from strangers.

#### 2.2.2. State-Level Predictors

**Gender equality measures.** In the present study, indicators for state policies related to gender equality included two indicators of reproductive rights (contraceptive access, abortion rights) and one indicator of economic equality between men and women.

***Reproductive rights.*** Reproductive rights/autonomy measures often incorporate indicators such as birth outcomes or life expectancy or adolescent birth rates [10,11] that could plausibly be related to the outcomes under consideration in this analysis. To avoid this potential limitation, we use two indicators of reproductive rights—indicators of the extent to which state policies facilitate or restrict contraceptive access and abortion rights—that more explicitly measure the extent to which women have control over their own reproductive lives [44].

*Contraceptive access* is a dichotomous measure indicating whether the state offered or required insurance coverage of contraception and/or low-income access to family planning, versus neither [44]. Of all respondents, 80.4% lived in states with some form of subsidized contraceptive access. States with restricted coverage of contraception were Alaska, Idaho, Kansas, Kentucky, Nebraska, North and South Dakota, Tennessee, Texas, Utah and Virginia.

*Abortion rights* scores from the year 2015 were derived from NARAL, a national pro-choice organization that tracks abortion and contraception policies in each state [44]. Each of the 50 states was assigned a grade from A+ to F. For the present analysis, states were dichotomized to reflect having more abortion rights (i.e., grade B− or higher) or fewer abortion rights. When women’s access to abortion is restricted, they may be at increased risk of intimate partner violence from a man involved in an unwanted pregnancy [45]. Of all respondents, just 43.7% lived in states with more abortion rights for women. States with more restricted access to abortion were Alabama, Arizona, Arkansas, Florida, Georgia, Idaho, Indiana, Kansas, Kentucky, Louisiana, Michigan, Mississippi, Missouri, Nebraska, North and South Carolina, North and South Dakota, Ohio, Oklahoma, Pennsylvania, Tennessee, Texas, Utah and Virginia.

***Economic equality.*** We used an existing measure of state-level economic equality between men and women that operationalizes economic equality using median annual earnings for women employed full-time year round, the earnings ratio between women and men employed full-time year round, percent of women in the labor force and percent of all women employed in managerial or professional occupations [46]. States were assigned a grade from A+ to F that reflected policies supporting economic equality between men and women. This measure was dichotomized into those states that support economic equality (i.e., grades ranging from A to C) versus not. Of all respondents, 78.7% lived in states supporting economic equality. States that scored poorly on this measure were Alabama, Arkansas, Florida, Idaho, Indiana, Kentucky, Louisiana, Mississippi, Montana, Nevada, Oklahoma, South Carolina, South Dakota, Utah and West Virginia.

**Drinking culture, by gender.** To operationalize drinking cultures, we used 2013 data from the Behavioral Risk Factors Surveillance System [47] on the percentage of men, and separately, women, in each state reporting binge drinking in the past 30 days. Binge drinking was defined as having four or five (or more) drinks on one occasion for women and men, respectively. The average state-level binge drinking rates across the US were 23.0% for men (SD = 2.2%) and 11.1% for women (SD = 2.2%). These data were centered on the overall US averages for the multivariate models. Higher rates of binge drinking indicate state drinking cultures more supportive of binge drinking. In the weighted analysis sample, state-level binge drinking rates for men and women were highly correlated (*r* = 0.87, *p* < 0.01).

**Median income.** Data on each state’s annual median household income came from the US American Community Survey, which is administered by the US Census Bureau [48]. The weighted average state-level median income for the analytic sample was US$54,006 (SD = $7768); the variable was transformed to represent increments of US$10,000. Median income was moderately positively correlated with men’s (*r* = 0.33, *p* < 0.01) and women’s (*r* = 0.44, *p* < 0.01) binge drinking rates, as well as with the measures of gender equality, with the weakest association being with the indicator of contraceptive access (*r* = 0.08, *p* < 0.01) and stronger associations with the indicators for abortion rights (*r* = 0.68, *p* < 0.01) and economic equality (*r* = 0.51, *p* < 0.01).

#### 2.2.3. Individual-Level Predictors

**Drinking status.** Each respondent’s past-year drinking status was derived from the National Institute on Alcohol Abuse and Alcoholism’s (NIAAA) stated guidelines for at-risk or heavy drinking. At-risk drinking is defined as consuming more than three or four drinks on a single day, or more than seven or 14 drinks per week, for women and men, respectively. Of the weighted analytic sample, 33.3% were non-drinkers, 38.9% were drinkers who did not exceed the recommended guidelines, and 27.8% were at-risk drinkers in the past year.

**Demographic covariates.** Models also adjusted for respondents’ gender, age, marital status (separated, divorced, widowed, and never married, versus married or living with a partner), race/ethnicity (using mutually-exclusive indicators for Hispanic, Black and other race/ethnicity, versus non-Hispanic White), education (less than a college degree versus a 4-year college degree or more), and income in the past year (classified into categories: “up to $20,000”, “$20,001–60,000”, “$60,001–$100,000”, “$100,001 or more”, and missing income, 11.6% of sample).

### 2.3. Analyses

The data consisted of a two-level hierarchy with individuals nested within states, and therefore we tested associations using multilevel logistic regression. We estimated main effects and interaction models using the state-level gender equality indicators and binge drinking rates by gender using the full sample, followed by gender-stratified analyses. For significant interactions, we also report Wald chi-square tests of the difference between the main effects and interaction models. Interactions were graphed using values of continuous variables at +/− 1 standard deviation from the mean. Data were weighted to adjust for the sampling strategy and nonresponse; all analyses were conducted using Stata version 15 [49].

## 3. Results

Descriptive statistics overall and for the gender-stratified samples are shown in Table 1. Women were older than men, and they also were more likely than men to be unmarried, Black and low-income. Women were more likely than men to be non-drinkers, and they were less likely than men to be at-risk drinkers. There were no differences by gender in any of the state-level variables.

### 3.1. Main Effects Models

In simultaneous main effects models testing the first hypothesis (1a), none of the gender equality measures were significantly associated with the second-hand harms due to alcohol overall, nor for men or women separately. These models also provide tests of the second hypothesis (1b): Adjusting for measures of gender equality and other state-level variables, the state rate of binge drinking by men was associated with increased odds of harms perpetrated by strangers overall (OR (95% CI) = 1.11 (1.00, 1.23)), although associations were not statistically significant in the gender-stratified models (OR (95% CI) = 1.12 (0.96, 1.31) for women; 1.09 (0.94, 1.26) for men). In the full sample, the association of male binge drinking rates with stranger-perpetrated harms persisted once individual-level covariates were added to the simultaneous model (OR (95% CI) = 1.12 (1.00, 1.24)). The male binge drinking rate was not significantly associated with the indicator of any past-year second-hand harm from alcohol, and it also was not significantly associated with spouse/partner-perpetrated harms. State rates of binge drinking by women and the state-level median household income were not significantly associated with second-hand harms from alcohol in any of the models.

### 3.2. Moderation Models

As expected (Hypothesis 2), there were some statistically significant interactions between indicators of gender equality and state male binge drinking rates. The findings for any past-year harm and for stranger-perpetrated harms suggested that the association between male binge drinking and second-hand harms was exacerbated by gender inequality.

#### 3.2.1. Any Past-Year Second-Hand Harm

**Figure 1:**
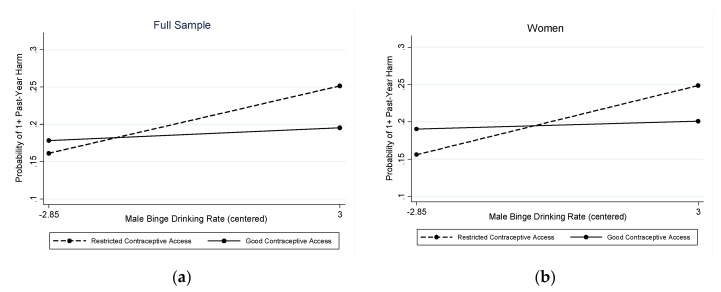
Interaction between male binge drinking rate and contraceptive access in relation to any second-hand harm from alcohol in the past year: (**a**) in the full sample (left panel); and (**b**) for women (right panel).

As shown in Table 2, in the full sample, there was a statistically significant interaction (OR = 0.92) between a state’s male binge drinking rate and contraceptive access in relation to any past-year second-hand harm due to someone else’s drinking (Wald chi-square (df = 18) = 441.69, *p* < 0.001). The marginal predicted probabilities showed a significant association of states’ male binge drinking rates with past-year harm due to someone else’s drinking only in states with restricted contraceptive access (the association was not significant in states with good contraceptive access): Predicted probabilities ranged from 0.161 (95% CI = 0.106, 0.216) at the lowest rates of binge drinking by men to 0.251 (95% CI = 0.187, 0.316) at the highest rates of binge drinking by men in states with restricted contraceptive access and from 0.178 (95% CI = 0.125, 0.231) at the lowest rates of binge drinking by men to 0.195 (95% CI = 0.136, 0.255) at the highest rates of binge drinking by men in states with good contraceptive access. The interaction is depicted in Figure 1 (left panel), which shows increasing rates of second-hand harms from alcohol as rates of men’s binge drinking increase, but only in states without good contraceptive access (top line, dashed).

The stratified models used to test Hypothesis 3 (Table 2 and Figure 1, right panel) revealed this pattern was unique to harms reported by women (OR = 0.91 for interaction and OR = 1.12 for the male binge drinking rate; Wald chi-square (df = 17) = 291.07, *p* < 0.001), with predicted probabilities of experiencing one or more harm due to someone else’s drinking ranging from 0.156 (95% CI = 0.113, 0.199) at the lowest rates of binge drinking by men to 0.249 (95% CI = 0.189, 0.309) at the highest rates of binge drinking by men in states with restricted contraceptive access and from 0.190 (95% CI = 0.149, 0.232) at the lowest rates of binge drinking by men to 0.200 (95% CI = 0.149, 0.253) at the highest rates of binge drinking by men in states with good contraceptive access. The interaction was not statistically significant for men, so it was dropped from the model. In the main effects model for men (Table 2), none of the state-level variables were significantly associated with past-year harms from someone else’s drinking.

#### 3.2.2. Past-Year Stranger-Perpetrated Harm

A similar pattern of results was found in the full sample when considering stranger-perpetrated harms. Both contraceptive access (OR = 0.89; Wald chi-square (df = 18) = 327.57, *p* < 0.001; Table 3) and abortion rights (OR = 0.90; Wald chi-square (df = 18) = 310.89, *p* < 0.001; Table 4) evidenced significant interactions with the male binge-drinking rate.

**Figure 2:**
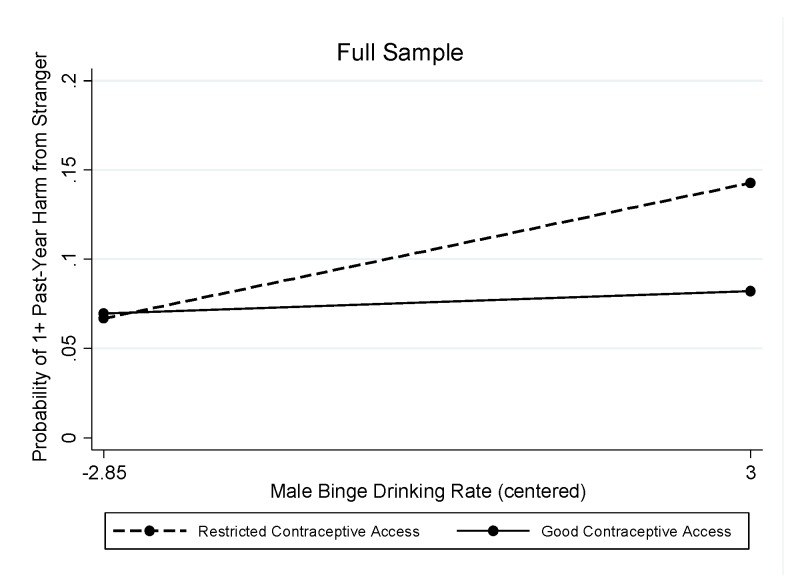
Interaction between male binge drinking rate and contraceptive access in relation to stranger-perpetrated harm in the full sample.

As shown in Table 3 and Figure 2, in states with restricted contraceptive access (the reference group), the male binge drinking rate was associated with significantly greater odds of harms from drinking strangers (OR = 1.17). The marginal predicted probabilities showed a significant association of states’ male binge drinking rates with past-year harm due to a stranger’s drinking only in states with restricted contraceptive access (the association was not significant in states with good contraceptive access): Predicted probabilities ranged from 0.067 (95% CI = 0.031, 0.103) at the lowest rates of binge drinking by men to 0.143 (95% CI = 0.096, 0.190) at the highest rates of binge drinking by men in states with restricted contraceptive access and from 0.070 (95% CI = 0.044, 0.096) at the lowest rates of binge drinking by men to 0.082 (95% CI = 0.051, 0.113) at the highest rates of binge drinking by men in states with good contraceptive access. In the stratified models, the interaction between contraceptive access and men’s binge drinking rates was not significant for either men or women, and in the main effect models (Table 3), none of the state-level variables were associated with harm from drinking strangers for either men or women.

**Figure 3:**
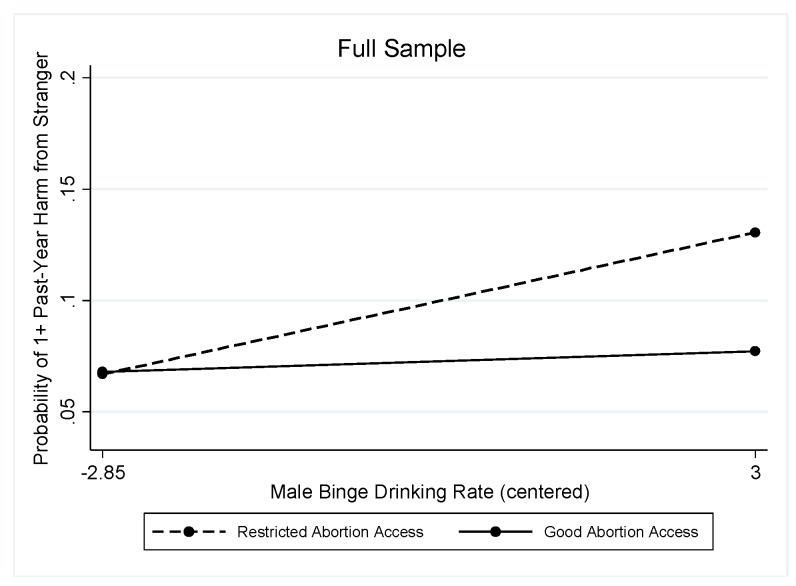
Interaction between the male binge drinking rate and abortion rights in relation to stranger-perpetrated harm in the full sample.

The interaction pattern was consistent when using the indicator of abortion rights. As shown in Table 4 and Figure 3, in states with restricted abortion access (the reference group), the male binge drinking rate was associated with significantly greater odds of harms from drinking strangers (OR = 1.14). The marginal predicted probabilities showed a significant association of states’ male binge drinking rates with past-year harm due to a stranger’s drinking only in states with restricted abortion access (the association was not significant in states with good abortion access): Predicted probabilities ranged from 0.067 (95% CI = 0.038, 0.095) at the lowest rates of binge drinking by men to 0.131 (95% CI = 0.088, 0.173) at the highest rates of binge drinking by men in states with restricted abortion access and from 0.068 (95% CI = 0.043, 0.093) at the lowest rates of binge drinking by men to 0.077 (95% CI = 0.043, 0.111) at the highest rates of binge drinking by men in states with good abortion access. In the stratified models, the interaction between abortion rights and male binge drinking rates was not significant for men, but it approached significance for women (OR = 0.88; Wald chi-square (df = 17) = 158.01, *p* < 0.001; Table 4).

#### 3.2.3. Past-Year Spouse- or Partner-Perpetrated Harm

**Figure 4:**
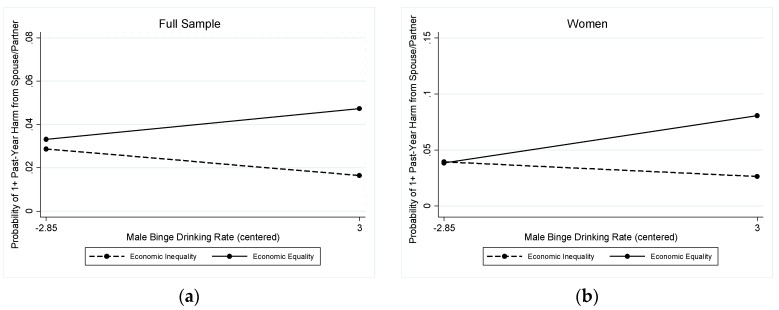
Interaction between male binge drinking rate and economic equality in relation to spouse-perpetrated harms (**a**) in the full sample (left panel); and (**b**) for women (right panel).

A different pattern of results emerged for spouse/partner-perpetrated harms. Only economic equality evidenced a significant interaction with the male binge-drinking rate (OR = 1.18; Wald chi-square (df = 18) = 166.53, *p* < 0.001; Table 5). The interaction is depicted in Figure 4 (left panel), which shows a diverging pattern, with higher rates of male binge drinking associated with only slightly higher rates of spouse/partner harm in states with economic equality (solid line) and somewhat decreasing rates of spouse/partner harm in states without economic equality (dashed line).

The marginal predicted probabilities suggested that states’ male binge drinking rates were not significantly associated with past-year harm due to a partner’s drinking in either group of states, however: Predicted probabilities ranged from 0.029 (95% CI = 0.015, 0.043) at the lowest rates of binge drinking by men to 0.016 (95% CI = 0.002, 0.031) at the highest rates of binge drinking by men in states without economic equality and from 0.033 (95% CI = 0.013, 0.054) at the lowest rates of binge drinking by men to 0.047 (95% CI = 0.017, 0.078) at the highest rates of binge drinking by men in states with economic equality. The stratified models revealed that this pattern was unique to harms reported by women (OR = 1.25; Wald chi-square (df = 17) = 79.34, *p* < 0.001; Table 5 and Figure 4, right panel), although the marginal predicted probabilities suggested that states’ male binge drinking rates were not significantly associated with women’s reports of past-year harm due to a partner’s drinking in either group of states: Predicted probabilities ranged from 0.039 (95% CI = 0.017, 0.062) at the lowest rates of binge drinking by men to 0.026 (95% CI = -0.004, 0.057) at the highest rates of binge drinking by men in states without economic equality and from 0.038 (95% CI = 0.011, 0.066) at the lowest rates of binge drinking by men to 0.081 (95% CI = 0.023, 0.138) at the highest rates of binge drinking by men in states with economic equality. The interaction was not statistically significant for men. In the main effects model for men (Table 5), none of the state-level variables were significantly associated with past-year harm due to a spouse or partner’s drinking.

## 4. Discussion

In this US study, with the exception of an association between male binge drinking and harms perpetrated by drinking strangers, we did not see any strong independent associations of either gender equality (Hypothesis 1a) or state-level drinking culture (Hypothesis 1b) with second-hand harms from alcohol. Our findings on Hypothesis 1a thus did not replicate prior studies showing an association between gender equality and reduced violence [8,30,31], suggesting the relationship between gender equality and alcohol-related harms is more complex than what can be captured by a main effect model. As hypothesized, we did find evidence that the combination of low gender equality (indexed here by indicators of restricted reproductive rights, including limited contraceptive access and curtailed abortion rights) and high levels of binge drinking by men was associated with increased odds of second-hand harms (Hypothesis 2). This relationship was seen in overall models and it was suggested in results for women, but not seen in results for men (Hypothesis 3). This moderation pattern held for an overall indicator of any second-hand harm in the past year, as well as for an indicator of harm attributed to drinking strangers. Thus, in this nationally-representative US sample, reproductive rights for women appear to buffer against the negative impacts of male binge drinking on others in the community, particularly on women. Future research should seek to replicate our findings, teasing out different types of alcohol-related harms at varying levels of severity, and to examine mechanisms through which these protective effects may occur, such as through changing social norms around violence or allowing women to not be tethered to violent men or to families or communities with high levels of violence.

When we examined alcohol-related harms perpetrated by intimate partners (spouses or boyfriends/ girlfriends), a different interaction pattern emerged. There were somewhat elevated rates of spouse/partner harm in states with economic equality but high levels of male binge drinking, but somewhat lower rates of spouse/partner harm in states without economic equality but high rates of male binge drinking. It may be that there is a backlash against women in these higher-equality, heavy-drinking states [8]. There also may be a tendency for people (women, in particular) in lower-equality, less-heavy-drinking states to label a partner’s drinking behavior as problematic, thus resulting in an increased reporting of harms in these contexts. Further, women in environments with greater gender equality may be more likely to divulge alcohol-related harms perpetrated by a partner or spouse. These possibilities should be examined in detail in longitudinal studies that could assess changes in gender equality and changes in second-hand harms from alcohol, as well as in qualitative studies that could describe the social construction of second-hand harms in regions with lower rates of binge drinking or higher economic equality.

We provide new evidence under Hypothesis 1b that a state-level drinking culture characterized by greater binge drinking by men is associated with second-hand harms from drinking strangers. Our findings are similar to prior research, showing that high levels of binge drinking on college campuses were related to reports of second-hand harms experienced by residents of nearby neighborhoods [38]. However, female drinking culture was not associated with second-hand alcohol harms, independently nor in tandem with measures of gender equality. This was not surprising given the contexts in which women drink in the US and other countries, with women tending to drink less often in public settings such as bars than men [32]. Drinking in public settings is associated with higher volume consumption and more opportunities for harms, both due to someone’s own drinking and from the drinking of people around them [50,51]. A future study could incorporate the drinking contexts in which these second-hand harms occur to examine differences across levels of gender equality. Furthermore, western drinking cultures generally endorse a normative view of high alcohol consumption among men, whereas women tend to be socialized otherwise [52,53]. However, in many age groups (with the exception of adolescents and young adults), heavy drinking among women in the US is steadily increasing [54] to meet rates comparable to those of men’s. Shifting drinking cultures may precede greater occurrences of second-hand harms from alcohol, with women’s drinking contributing more to these harms as women’s drinking cultures continue to evolve. Intersection of heavy drinking by women with lower levels of gender equality also may put women at greater risk of alcohol-related harm.

To our knowledge, this is one of the first US studies on the topic of state-level gender equality, drinking cultures and alcohol’s harm to others. Although we had the benefit of a large, nationally-representative sample of adult respondents, our data were cross-sectional, and causality cannot be implied. Although it is possible that reductions in the second-hand effects of alcohol could precede changes in gender equality, it is less likely that changes in these harms would reduce binge drinking by men or prompt legislators to enact laws and policies protecting women’s reproductive rights. Thus, it seems reasonable to assume the outcomes studied here may have been at least partially a result of the policy context and drinking culture of the respondents’ state of residence. Stronger causal models would enhance the evidence base on this topic.

Another study limitation is that there may be other important effect modifiers that we did not take into account, such as a respondent’s level of education [12] or work status [55], which have been shown to interact with gender equality in studies of drinking behavior. An additional consideration is that some of the US states with the lowest gender equality (such as Idaho, South Dakota and Utah) are also relatively sparsely populated, and may be under-represented in general population samples such as ours. 

Further, the indicators of gender equality are not independent, and isolation of their effects may be impossible. For example, four states scored low on all three measures of gender equality, and fourteen states scored low on two of the three measures. Future work may benefit from the utilization of summary scores that better describe the status of women on multiple dimensions. Finally, additional work is needed to disentangle the effects of gender equality, drinking cultures and attitudes toward violence (and particularly violence to women) on alcohol’s second-hand harms, as these may be related in complex ways [56]. Future work also should examine overall cultures of violence, using indicators such as levels of violence toward women or state laws on domestic violence and sexual assault, in relation to second-hand harms from alcohol.

## 5. Conclusions

Findings from a large, population-representative study of US adults suggest detrimental effects of high rates of male binge drinking may be exacerbated by gender inequality, with this effect being particularly harmful for women. As reproductive rights decrease in U.S. states [57], policy makers in states with high levels of binge drinking should be aware that changes in reproductive rights might increase second-hand harms from alcohol experienced by women in these states.

## Figures and Tables

**Table 1:** Sample descriptives and weighted bivariate comparisons by gender (*N* = 7792).

Variables	Full Sample	Men	Women	*p*
	*N* = 7792	*n* = 3156	*n* = 4636	
Unmarried (%)	42.3	40.1	44.3	*
Race/Ethnicity (%)				***
White	66.4	67.2	65.8	
Black	11.9	10.7	13.0	
Hispanic	14.1	15.0	13.3	
Other/Missing	7.5	7.1	7.9	
Less than College Degree	70.2	69.1	71.2	
Income (%)				***
Up to $20,000	20.9	18.0	23.6	
$20,000–$60,000	32.6	33.7	31.7	
$60,001–$100,000	20.3	21.0	19.7	
$100,001 or more	14.9	18.0	12.1	
Missing Income	11.3	9.3	13.0	
Age, M (SD)	52.6 (17.7)	51.3 (17.6)	53.5 (17.6)	^†^
Drinking Status (%)				***
Non-Drinker	33.3	29.7	36.7	
Drinker, Does not Exceed Guidelines ^a^	38.8	37.4	40.0	
At-risk Drinker, Exceeds Guidelines	28.0	32.9	23.3	
State Median Income (by $10k), M (SD)	5.4 (0.8)	5.5 (0.8)	5.5 (0.8)	
State Male Binge Drinking %, M (SD)	23.1 (2.9)	23.0 (2.9)	23.0 (2.9)	
State Female Binge Drinking %, M (SD)	11.1 (2.1)	11.1 (2.1)	11.0 (2.1)	
State Gender Equality Indicators (%)				
Contraceptive Access	80.5	80.3	80.6	
Reproductive Rights	43.6	44.2	43.1	
Economic Equality	78.7	79.0	78.4	

*** *p* < 0.001 * *p* < 0.05 ^†^ *p* < 0.10; ^a^ U.S. National Institute on Alcohol Abuse and Alcoholism’s (NIAAA) guidelines define at-risk drinking as consuming more than 3 or 4 drinks on a single day, or more than 7 or 14 drinks per week, for women and men, respectively.

**Table 2:** Interactive associations of states’ male binge drinking rates and contraceptive access with any second-hand harm from others’ drinking ^1^.

Predictor Variables	Full Sample	Men	Women
	(*N* = 7792)	(*n* = 3156)	(*n* = 4636)
	OR (95% CI)	OR (95% CI)	OR (95% CI)
Male	0.93 (0.80, 1.08)	-	-
Unmarried	1.36 (1.06, 1.74) *	1.71 (1.26, 2.31) ***	1.13 (0.87, 1.48)
Race/Ethnicity ^2^			
Black	1.06 (0.85, 1.31)	1.06 (0.70, 1.59)	1.03 (0.78, 1.37)
Hispanic	0.87 (0.71, 1.05)	0.96 (0.62, 1.49)	0.74 (0.53, 1.03) ^†^
Other/Missing	1.40 (0.99, 2.00) ^†^	1.69 (1.06, 2.69) *	1.21 (0.71, 2.07)
Less than College Degree	1.08 (0.86, 1.36)	1.20 (0.84, 1.69)	1.02 (0.74, 1.39)
Income ^3^			
Up to $20,000	1.75 (1.26, 2.43) **	1.42 (0.90, 2.26)	2.05 (1.37, 3.08) ***
$20,000–$60,000	1.41 (1.04, 1.93) *	1.28 (0.76, 2.15)	1.55 (1.06, 2.27) *
$60,001–$100,000	1.11 (0.81, 1.52)	1.21 (0.75, 1.94)	0.96 (0.62, 1.48)
Missing Income	0.99 (0.74, 1.33)	1.09 (0.62, 1.95)	0.90 (0.57, 1.41)
Age	0.98 (0.97, 0.98) ***	0.98 (0.97, 0.99) ***	0.97 (0.96, 0.98) ***
Drinking Status ^4^			
Drinker, Does not Exceed Guidelines	1.14 (0.90, 1.45)	0.96 (0.64, 1.44)	1.26 (0.92, 1.74)
At-risk Drinker, Exceeds Guidelines	2.32 (1.89, 2.84) ***	2.14 (1.51, 3.03)	2.53 (1.86, 3.44) ***
State Median Income	1.06 (0.17, 6.65)	0.92 (0.05, 16.82)	1.97 (0.88, 1.17)
State Male Binge Drinking Rate	1.11 (1.01, 1.23) *	1.07 (0.93, 1.24)	1.12 (1.02, 1.22) *
State Female Binge Drinking Rate	1.02 (0.86, 1.22)	1.00 (0.79, 1.25)	1.01 (0.88, 1.17)
Contraceptive Access ^5^	0.90 (0.65, 1.24)	0.84 (0.49, 1.43)	0.99 (0.73, 1.32)
Male Binge * Contraceptive Access ^6^	0.92 (0.85, 0.99) *	-	0.91 (0.85, 0.97) **
Constant	0.34 (0.12, 0.96) *	0.23 (0.03, 1.60)	0.29 (0.12, 0.69) **

Note: *** *p* < 0.001 ** *p* < 0.01 * *p* < 0.05 ^†^ *p* < 0.10; ^1^ Harm from Others’ Drinking = Reported one or more of 8 second-hand harms in the past year; ^2^ Reference = White; ^3^ Reference = $100,001+; ^4^ Reference = Non-drinker; ^5^ Contraceptive access = The state offered or required insurance coverage of contraception and/or low-income access to family planning, versus neither; ^6^ Main effects model reported for men because interaction was not significant, *p* > 0.10.

**Table 3:** Interactive associations of male binge drinking rates and contraceptive access with harm from drinking strangers.

Predictor Variables	Full Sample	Men	Women
	(*N* = 7792)	(*n* = 3156)	(*n* = 4636)
	OR (95% CI)	OR (95% CI)	OR (95% CI)
Male	1.37 (1.07, 1.74) *	-	-
Unmarried	1.34 (1.03, 1.75) *	1.52 (0.96, 2.40) ^†^	1.31 (0.92, 1.87)
Race/Ethnicity ^1^			
Black	1.08 (0.74, 1.57)	1.19 (0.67, 2.11)	0.92 (0.57, 1.47)
Hispanic	0.90 (0.66, 1.21)	0.86 (0.51, 1.46)	0.82 (0.44, 1.51)
Other/Missing	1.64 (1.11, 2.42) *	2.49 (1.43, 4.33) **	0.97 (0.48, 1.97)
Less than College Degree	0.90 (0.64, 1.26)	1.21 (0.74, 1.98)	0.67 (0.43, 1.05) ^†^
Income ^2^			
Up to $20,000	1.34 (0.85, 2.11)	0.84 (0.43, 1.63)	1.89 (0.91, 3.96) ^†^
$20,000–$60,000	1.23 (0.75, 2.04)	1.21 (0.60, 2.44)	1.19 (0.65, 2.16)
$60,001–$100,000	0.97 (0.64, 1.48)	1.04 (0.53, 2.04)	0.82 (0.38, 1.77)
Missing Income	1.01 (0.61, 1.67)	1.08 (0.52, 2.22)	0.88 (0.36, 2.14)
Age	0.97 (0.97, 0.98) ***	0.99 (0.97, 1.00) *	0.96 (0.95, 0.97) ***
Drinking Status ^3^			
Drinker, Does not Exceed Guidelines	0.95 (0.70, 1.29)	0.89 (0.55, 1.43)	0.92 (0.53, 1.61)
At-risk Drinker, Exceeds Guidelines	1.85 (1.41, 2.44) ***	1.80 (1.11, 2.93) *	1.82 (1.06, 3.12) *
State Median Income	1.02 (0.15, 7.11)	0.48 (0.02, 12.16)	1.72 (0.74, 1.18)
State Male Binge Drinking Rate	1.17 (1.04, 1.31) *	1.08 (0.94, 1.25)	1.13 (0.95, 1.36)
State Female Binge Drinking Rate	1.02 (0.85, 1.22)	1.02 (0.80, 1.28)	0.93 (0.74, 1.18)
Contraceptive Access ^4^	0.74 (0.48, 1.14)	0.84 (0.38, 1.87)	0.68 (0.35, 1.34)
Male Binge * Contraceptive Access ^5^	0.89 (0.81, 0.97) *	-	-
Constant	1.73 (0.44, 0.67) *	0.13 (0.01, 1.32) ^†^	0.32 (0.06, 1.76)

Note: *** *p* < 0.001 ** *p* < 0.01 * *p* < 0.05 ^†^ *p* < 0.10; ^1^ Reference = White; ^2^ Reference = $100,001+; ^3^ Reference = Non-drinker; ^4^ Contraceptive access = The state offered or required insurance coverage of contraception and/or low-income access to family planning, versus neither; ^5^ Main effects model reported for gender-stratified analyses because interactions were not significant, *p* > 0.10.

**Table 4:** Interactive associations of male binge drinking rates and abortion rights with harm from drinking strangers.

Predictor Variables	Full Sample	Men	Women
	(*N* = 7792)	(*n* = 3156)	(*n* = 4636)
	OR (95% CI)	OR (95% CI)	OR (95% CI)
Male	1.36 (1.07, 1.74) *	-	-
Unmarried	1.34 (1.03, 1.74) *	1.52 (0.96, 2.41) ^†^	1.31 (0.92, 1.86)
Race/Ethnicity ^1^			
Black	1.08 (0.74, 1.56)	1.18 (0.67, 2.08)	0.90 (0.56, 1.44)
Hispanic	0.90 (0.66, 1.22)	0.87 (0.51, 1.48)	0.81 (0.44, 1.49)
Other/Missing	1.63 (1.11, 2.41) *	2.50 (1.44, 4.35) **	0.97 (0.47, 1.97)
Less than College Degree	0.90 (0.64, 1.26)	1.21 (0.74, 1.97)	0.67 (0.43, 1.05) ^†^
Income ^2^			
Up to $20,000	1.34 (0.85, 2.11)	0.84 (0.43, 1.63)	1.90 (0.90, 3.99) ^†^
$20,000–$60,000	1.23 (0.75, 2.03)	1.21 (0.60, 2.42)	1.19 (0.65, 2.16)
$60,001–$100,000	0.97 (0.64, 1.48)	1.04 (0.53, 2.03)	0.81 (0.37, 1.76)
Missing Income	1.01 (0.61, 1.67)	1.08 (0.52, 2.22)	0.89 (0.36, 2.16)
Age	0.97 (0.97, 0.98) ***	0.99 (0.97, 1.00) *	0.96 (0.95, 0.97) ***
Drinking Status ^3^			
Drinker, Does not Exceed Guidelines	0.95 (0.70, 1.29)	0.89 (0.55, 1.42)	0.91 (0.52, 1.59)
At-risk Drinker, Exceeds Guidelines	1.85 (1.40, 2.43) ***	1.80 (1.11, 2.92) *	1.81 (1.05, 3.11) *
State Median Income	1.68 (0.26, 11.02)	1.63 (0.07, 37.35)	2.87 (0.39, 20.94)
State Male Binge Drinking Rate	1.14 (1.02, 1.29) *	1.06 (0.92, 1.23)	1.18 (0.95, 1.48)
State Female Binge Drinking Rate	1.02 (0.85, 1.22)	1.07 (0.83, 1.38)	0.96 (0.74, 1.25)
Abortion Rights	0.74 (0.49, 1.12)	0.64 (0.30, 1.37)	0.81 (0.46, 1.42)
Male Binge * Abortion Rights ^4^	0.90 (0.82, 0.98) *	-	0.88 (0.76, 1.01) ^†^
Constant	0.12 (0.04, 0.40) ***	0.07 (0.01, 0.51) **	0.21 (0.54, 0.84) *

Note: *** *p* < 0.001 ** *p* < 0.01 * *p* < 0.05 ^†^*p* < 0.10; ^1^ Reference = White; ^2^ Reference = $100,001+; ^3^ Reference = Non-drinker; ^4^ Main effects model reported for men because interaction was not significant, *p* > 0.10.

**Table 5:** Interactive associations of male binge drinking rates and economic equality with harm from drinking spouse/partner.

Predictor Variables	Full Sample	Men	Women
	(*N* = 7792)	(*n* = 3156)	(*n* = 4636)
	OR (95% CI)	OR (95% CI)	OR (95% CI)
Male	0.42 (0.26, 0.68) ***	-	-
Unmarried	0.87 (0.56, 1.34)	1.02 (0.38, 2.69)	0.84 (0.50, 1.42)
Race/Ethnicity ^1^			
Black	0.96 (0.52, 1.79)	1.49 (0.57, 3.88)	0.80 (0.39, 1.65)
Hispanic	0.78 (0.45, 1.35)	1.38 (0.44, 4.34)	0.66 (0.34, 1.30)
Other/Missing	1.71 (0.87, 3.39)	3.02 (0.90, 10.15) †	1.40 (0.61, 3.23)
Less than College Degree	1.50 (0.98, 2.31) ^†^	1.29 (0.52, 3.20)	1.53 (0.96, 2.44) ^†^
Income ^2^			
Up to $20,000	1.81 (0.94, 3.45) ^†^	1.90 (0.71, 5.07)	1.87 (0.76, 4.61)
$20,000–$60,000	1.20 (0.67, 2.17)	0.66 (0.20, 2.24)	1.60 (0.69, 3.68)
$60,001–$100,000	0.95 (0.49, 1.85)	0.80 (0.38, 1.69)	0.98 (0.41, 2.34)
Missing Income	0.58 (0.25, 1.38)	0.77 (1.17, 3.53)	0.49 (0.18, 1.31)
Age	0.98 (0.97, 0.99) ***	1.00 (0.98, 1.01)	0.97 (0.96, 0.98) ***
Drinking Status ^3^			
Drinker, Does not Exceed Guidelines	1.51 (0.88, 2.58)	1.64 (0.53, 5.06)	1.39 (0.67, 2.88)
At-risk Drinker, Exceeds Guidelines	2.23 (1.37, 3.65) **	2.68 (1.04, 6.88) *	2.13 (1.06, 4.27) *
State Median Income	2.96 (0.07, 117.38)	7.06 (0.06, 781.29)	5.16 (0.05, 540.02)
State Male Binge Drinking Rate	0.90 (0.76, 1.07)	0.90 (0.71, 1.14)	0.93 (0.74, 1.16)
State Female Binge Drinking Rate	0.90 (0.67, 1.20)	1.02 (0.68, 1.50)	0.85 (0.61, 1.19)
Economic Equality	1.88 (0.97, 3.63) ^†^	1.39 (0.62, 3.12)	1.83 (0.70, 4.80)
Male Binge * Economic Equality ^4^	1.18 (1.00, 1.39) *	-	1.25 (1.02, 1.54) *
Constant	0.02 (0.00, 0.12) ***	0.00 (0.00, 0.03) ***	0.01 (0.00, 0.16) **

Note: *** *p* < 0.001 ** *p* < 0.01 * *p* < 0.05 ^†^ *p* < 0.10; ^1^ Reference = White; ^2^ Reference = $100,001+; ^3^ Reference = Non-drinker; ^4^ Main effects model reported for men because interaction was not significant, *p* > 0.10.
